# The impact of genome-wide association studies on biomedical research publications

**DOI:** 10.1186/s40246-018-0172-4

**Published:** 2018-08-13

**Authors:** Travis J. Struck, Brian K. Mannakee, Ryan N. Gutenkunst

**Affiliations:** 1 Department of Molecular and Cellular Biology, University of Arizona, Tucson, AZ USA; 2 Department of Epidemiology and Biostatistics, Mel and Enid Zuckerman College of Public Health, University of Arizona, Tucson, AZ USA

**Keywords:** Genome-wide association studies, Bibliometrics, Follow-up research

## Abstract

The past decade has seen major investment in genome-wide association studies (GWAS). Among the many goals of GWAS, a major one is to identify and motivate research on novel genes involved in complex human disease. To assess whether this goal is being met, we quantified the effect of GWAS on the overall distribution of biomedical research publications and on the subsequent publication history of genes newly associated with complex disease. We found that the historical skew of publications toward genes involved in Mendelian disease has not changed since the advent of GWAS. Genes newly implicated by GWAS in complex disease do experience additional publications compared to control genes, and they are more likely to become exceptionally studied. But the magnitude of both effects has declined over the past decade. Our results suggest that reforms to encourage follow-up studies may be needed for GWAS to most successfully guide biomedical research toward the molecular mechanisms underlying complex human disease.

**Electronic supplementary material:**

The online version of this article (10.1186/s40246-018-0172-4) contains supplementary material, which is available to authorized users.

## Background

Since the first successful genome-wide association studies (GWAS) were published over a decade ago [1–4], thousands have been performed [5]. These studies have identified tens of thousands of statistical associations between genetic variants and human diseases [5]. The large investment in GWAS has been criticized [6], perhaps because initial hopes for quick clinical impact were overenthusiastic [7]. The average time from basic science discovery to clinical practice is 17 years [8], so it is unsurprising that few GWAS results directly affect patients yet. But direct clinical impact is not the only goal of GWAS.

One major goal of GWAS has been to broadly characterize the genetic basis of human traits and complex disease. GWAS have shown that most traits are highly polygenic and that most common variants exhibit small effect size on phenotype [9, 10]. They have also shown that genetic variants associated with disease are strongly enriched in regulatory regions [11] and that pleiotropy is pervasive [12, 13]. They have also enabled polygenic prediction of traits by aggregating the weak effects of many variants [14, 15], although not yet with clinical precision [16]. These insights have motivated a number of large public genomics projects, such as the ENCODE project to identify functional genomic elements [17], the Epigenome Roadmap project to identify tissue-specific epigenomic regulation [18], the GTEx project to connect genetic variation with tissue-specific gene expression [19], and the Human Cell Atlas project to identify and characterize all cell types in the body [20].

Another major goal of GWAS has been to specifically identify novel genes involved in complex disease and steer research toward them [16, 21, 22]. Identifying the causal genetic variant and the affected gene(s) that drive an association can be challenging [23], but integrating data from large genomics projects can provide important clues [24]. Novel connections between genes and diseases can lead to new treatments. For example, an early GWAS unexpectedly found variation in complement factor H to be strongly associated with macular degeneration [2], spurring the development of complement-based therapeutics [25]. Similarly, associations between variation in the interleukin-23 receptor and Crohn’s disease [26] and psoriasis [27] motivated the development of several treatments that are now in clinical trials [28]. In both of these classic examples, going from association to therapy demanded substantial follow-up research.

Beyond anecdotal examples, how much follow-up research typically occurs when a gene is newly associated with complex disease via GWAS? To answer this question, we assessed the impact of GWAS on subsequent biomedical research publications. Our motivation was that if there is little follow-up research on associated genes, then important medical innovations are possibly being missed, and reforms may be necessary to encourage follow-up research.

Published GWAS are themselves often highly cited, for example [4, 26, 29]. A systematic comparison also found that GWAS are more highly cited than comparable candidate gene studies [30]. But a paper that cites a GWAS does not necessarily follow-up on the associations reported by that GWAS. To quantify how much follow-up research is motivated by GWAS, we focused on the subsequent publication record of newly associated genes.

**Fig. 1:**
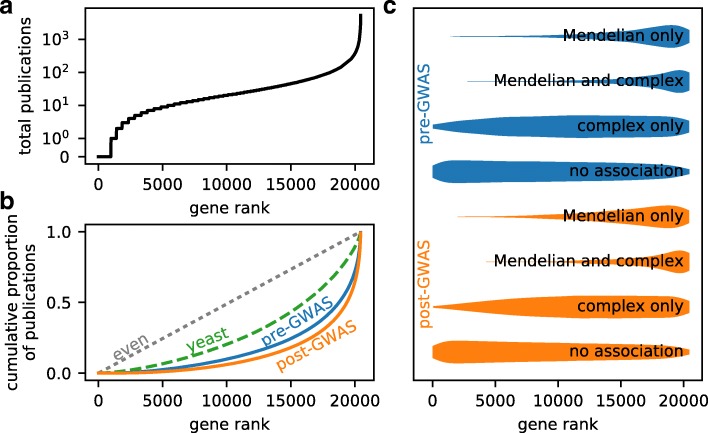
Biomedical scientific publications are highly unequally distributed and strongly skewed toward genes involved in Mendelian disease, even after the advent of GWAS. **a** The distribution of publications among all human genes is highly uneven. Plotted is the number of publications per gene, with genes sorted by number of publications. (The gene with the fewest publications is plotted as rank 1, and the gene with the most publications as rank 20,422.) A few genes are the subject of thousands of publications each, whereas thousands of genes are the subject of fewer than ten publications each. **b** The distribution of publications among all human genes is more uneven in the post-GWAS era (2005 and later) than in the pre-GWAS era (before 2005). Shown in this Gini plot are the cumulative proportions of publications in each category versus gene rank. The further the curve is from the diagonal, the more uneven the distribution. For comparison, the distribution of publications among yeast genes is shown, with the yeast *x*-axis stretched to match the number of human genes. **c** Highly studied genes tend to be involved in Mendelian disease. Plotted are the distributions of genes among publication rank for genes of each possible type of disease association and for both the pre- and post-GWAS eras. (Distributions are not normalized across types of disease association.) In both eras, genes involved in Mendelian diseases are strongly enriched toward high publication ranks. By contrast, many genes involved only in complex disease rank low in terms of publications

The distribution of biomedical research publications is highly unequal among human genes (Fig. 1a; [31]). Much of this inequality stems from historical momentum, driven by the availability of prior functional information [32] or research tools [33]. Consequently, many potentially medically important genes may be understudied [34]. Because GWAS are largely unbiased by previous knowledge about genes [35], they provide an opportunity for understudied genes to be brought to the scientific forefront.

We evaluated the effect of GWAS on the biomedical research literature in three ways. At a broad scale, we tested whether the distribution of publications among human genes has changed since the advent of GWAS. At a narrower scale, we quantified the effect of being newly associated with complex disease on the subsequent publication histories of human genes. Lastly, we identified outlier genes with exceptional publication activity and tested whether GWAS might play a role in motivating such activity. Overall, we find that genes newly associated with complex disease do experience increases in publication activity, but this effect has declined over the past decade.

## Results

We measured research output on genes using scientific publications, as collected in the NCBI Gene database [36]. We prefer this manually curated database to automatic text mining, because text mining may introduce false positives when a gene is mentioned in passing. In total, we considered 553,184 biomedical research publications that appeared in the annotations for one or more human genes, most of which were published after 1995 (Additional file 1: Figure S1).

### Broad patterns of publications on human genes

We used the Online Mendelian Inheritance in Man (OMIM) database [37] and the EBI-NCBI GWAS catalog [5] to classify genes into those associated with Mendelian disease (*N* =1126), complex disease (*N* =3648), both (*N* =595), or no disease (*N* =15,043). As expected [31], we found that the distribution of publications among human genes was highly uneven. A small number of genes were the subject of many thousands of publications, while a large number of genes were the subject of only a few (Fig. 1a).

To quantify the unevenness of publications among genes, we used the Gini coefficient, which ranges from 0 (perfectly even distribution) to 1 (perfectly uneven). The Gini coefficient is calculated from the cumulative distribution of publications versus the gene rank (Fig. 1b). To quantify the effect of GWAS on the distribution of publications among human genes, we compared that distribution before and after 2005. We chose 2005 as the cutoff between pre- and post-GWAS eras, because that is the year of the first entry in the GWAS catalog [5]. Other appropriate cutoff years might be 2007, when the first large GWAS were published, or 2009, to give time for publication patterns to change. Using either of these cutoff years does not qualitatively change our results (Additional file 1: Figure S2). The inequality of publications among human genes is larger in the post-GWAS era than in the pre-GWAS era (Gini coefficient 0.73 vs 0.65; Fig. 1b). It is not inevitable that the distribution of publications should be so unequal; the Gini coefficient of publications among yeast genes is much lower at 0.43 (Fig. 1b).

The ultimate goal of most biomedical research is to improve human health, so the distribution of publications is expected to be skewed toward genes involved in human disease. In the pre-GWAS era, genes associated with Mendelian disease were, almost without exception, among the most highly studied human genes (Fig. 1c and Additional file 1: Figure S2). By contrast, many genes that would later be associated with complex disease were among the least studied human genes (Fig. 1c). The advent of GWAS led to the discovery of many genes associated with complex human disease. The focus of biomedical publications on Mendelian disease genes, however, remains strong in the post-GWAS era (Fig. 1c). In particular, many genes associated with complex disease remain among the least studied genes in the human genome (Fig. 1c). The distribution of publication ranks for genes associated only with complex disease has shifted slightly toward higher ranks in the post-GWAS era compared to the pre-GWAS era (Mann-Whitney *U* test, *p*∼10^−9^, *N* =3648), but the distribution has not changed qualitatively. Examining the distributions of publication ranks at higher temporal resolution also does not reveal any qualitative changes (Additional file 1: Figure S3).

### Subsequent publications on individual genes

**Fig. 2:**
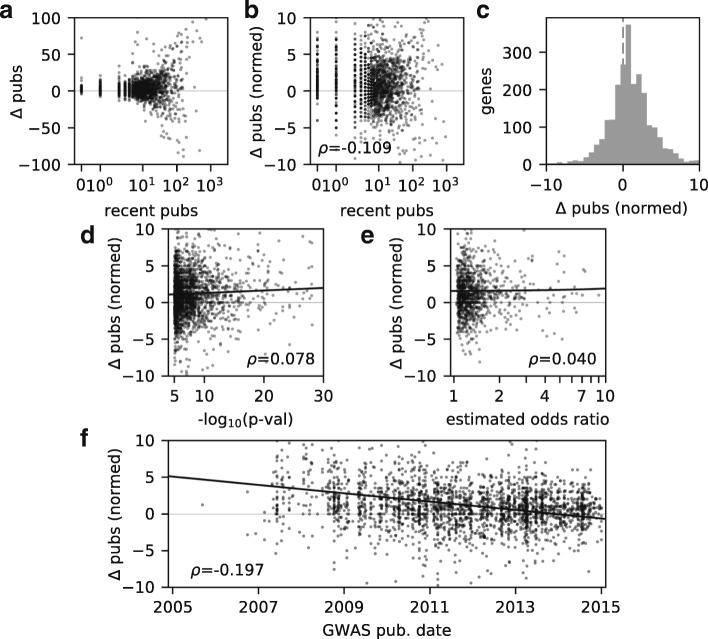
Effect on subsequent publications for genes newly associated with complex disease via GWAS. To quantify the short-term effects of GWAS association, we considered the publication excess of each newly associated gene compared with its control gene. **a** The variance of the publication excess is strongly correlated with the associated gene’s number of recent publications. **b** Normalizing the publication excess by the square root of the number of recent publications equalizes the variance. It also reveals a trend for the normalized effect of GWAS association to be smaller for more heavily studied genes. **c** The distribution of normalized publication excess is shifted toward positive values, indicating a positive effect of GWAS association on subsequent publications. **d** The normalized publication excess for a newly associated gene is weakly correlated with the *p* value of the association. **e** It is not statistically significantly correlated with the estimated effect size of the association, as quantified by the reported odds ratio. **f** The normalized publication excess is negatively correlated with the publication date of the association. More recently associated genes experience a smaller increase in subsequent publications. Reported correlations *ρ* are Spearman rank correlations, and thick black lines in panels **d**–**f** are linear regressions

To quantify the immediate effect of GWAS on research into individual newly associated genes, we considered all genes that were first associated with complex disease via GWAS before 2015 (*N* =2442), and we focused on the calendar year of the first association and the following 2 years. For each new GWAS gene, we compared the publications over this period with a control non-GWAS gene chosen to have as similar a prior publication history as possible (see the “Materials and methods” Section). The variance in an associated gene’s publications is strongly correlated with the number of publications on that gene in the prior 3 years (Fig. 2a). Normalizing the excess in publications relative to the control gene by the square root of the number of recent publications normalizes the variance (Fig. 2b), consistent with a Poisson model for publication output [38]. The normalized excess in publications for a GWAS gene is slightly but significantly shifted (Fig. 2c; one-sample *t* test, *p*∼5×10^−34^, *N* =2442). The mean normalized excess is 1.24 units, corresponding to a mean excess of 2.95 publications over the 3 years following association.

We next sought to identify the factors that determine how large an effect a GWAS will have on an associated gene’s subsequent publications. For example, the more heavily studied a gene was previously, the smaller the effect of GWAS association (Fig. 2b, Spearman rank correlation, *p*∼6×10^−8^, *N* = 2442).

The strength of a GWAS association is quantified by its statistical *p* value and its estimated biological effect size, which is most commonly an odds ratio. The normalized publication excess for a newly associated gene is weakly positively correlated with the *p* value of its association (Fig. 2d; *p*∼1×10^−4^, *N* =2442). By contrast, the normalized publication excess is not significantly correlated with the estimated effect size of the reported association (Fig. 2e; *p*∼0.14, *N* =1327).

The strongest predictor of the effect of a GWAS on future publications for associated genes is the year in which the GWAS was published. The typical normalized publication excess has declined dramatically since the early years of GWAS (Fig. 2f; *p*∼9×10^−23^,*N*=2442).

The predictors for the effect of GWAS on subsequent publications that we have studied may themselves be correlated; to disentangle their effects, we built a linear regression model. In that model, the effects of the number of recent publications and GWAS publication date are strong and statistically significant (Table 1). By contrast, the quantitative properties of the association itself, the *p* value and the estimated effect size, have weak effects that are not statistically significant.

**Table 1:** Linear regression model for the normalized publication excess of new GWAS genes (*N*=1232)

Predictor	Coefficient	Std. error	*p* value
log10(recent pubs)	−0.741	0.281	0.008
−log10(*p* value)	0.032	0.018	0.083
Estimated odds ratio	0.032	0.061	0.501
GWAS pub. date	−0.730	0.078	<10^−19^

The GWAS catalog uses a relatively liberal *p* value threshold of 10^−5^ for inclusion of associations into the catalog, and large *p* value associations may be statistical noise that subsequent researchers properly ignore. To account for this effect, we repeated our analyses using only genes for which the first reported association had *p*<10^−8^, the suggested threshold for testing low-frequency variants [39]. When we restricted our analysis to these high-confidence associations (Additional file 1: Figure S4), we found that normalized publication excess was no longer significantly correlated with *p* value (*ρ*=0.044,*p*∼0.23; *N*=724), but it was positively correlated with estimated effect size (*ρ*=0.094; *p*∼0.025; *N*=570). The negative correlation between normalized publication excess and GWAS publication date was stronger than in the full data (*ρ*=−0.33; *p*∼7×10^−20^). The linear regression model (Additional file 1: Table S1) was similar to the full data, with the effects that were statistically not significant for *p* value and estimated effect size and significant for number of recent publications and GWAS publication date. Further restricting our analysis to associations for which the lower bound of the 95% confidence interval on the estimated odds ratio was larger than 1.1 (Additional file 1: Figure S5) yielded qualitatively similar results (Additional file 1: Figure S6 and Table S2).

Association with particular diseases might lead to particularly intense study. To test this possibility, we considered the class of disease that each gene was associated with as an additional predictor in the linear regression model. Of the 20 disease classes tested, only metabolic disease had a significant effect on the normalized publication excess (Additional file 1: Table S3). Further stratifying among metabolic diseases, we found that this trend is driven by studies on type II diabetes and obesity (Additional file 1: Table S4).

### Genes with exceptional publication records

**Fig. 3:**
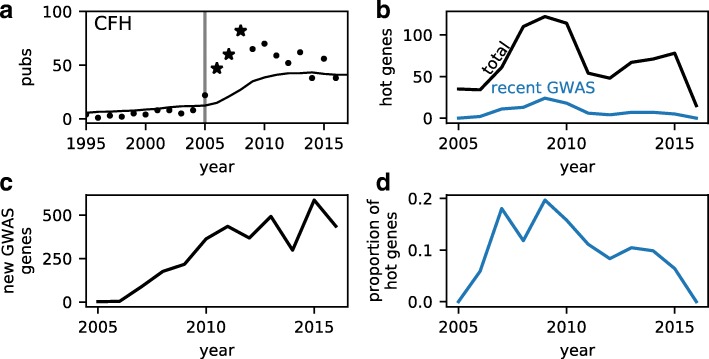
The effect of GWAS in generating exceptionally studied genes. **a** A significantly elevated number of studies were published on complement factor H following its association with macular degeneration via GWAS in 2005 [2]. Solid line is the predicted publication history from the model of Pfeiffer and Hoffmann [38], points indicate actual publication counts, and starred points indicate years with a statistically significant excess (one-sided Bonferroni-corrected *p*<0.05). **b** The total number of genes exhibiting an unusual excess in publications peaked in 2009, as did the number of those genes that were recently newly associated with complex disease via GWAS. **c** The number of genes newly associated with complex disease through GWAS has grown since the inception of GWAS. **d** The proportion of genes exhibiting an unusual excess in publications that were recently identified in GWAS peaked at roughly 20% in 2009 and has since declined

The typical new GWAS gene experiences a modest increase in subsequent publications, but some exceptional genes may experience large increases, so-called hot genes. To identify such genes, we used the model of Pfeiffer and Hoffmann [38] to predict the number of publications for each gene in each year, based on that gene’s prior publication history. We trained the model on all genes never implicated in complex disease through GWAS. By comparing the model predictions and publication data, we then identified particular years in which particular genes had unexpectedly large numbers of publications (Additional file 2). For example, complement factor H had a significant excess of publications in all 3 years following its association with macular degeneration (Fig. 3a).

The total number of hot genes per year has recently fluctuated (Fig. 3b). Between 2009 and 2016, on average, 0.3% of genes were hot in any given year. Of the genes that were newly associated with complex disease via GWAS within the past 3 years, the probability of being hot was 1.3%. So, being newly associated with complex disease does increase the probability that a gene will become hot. The total number of hot genes that were recently associated with complex disease via GWAS peaked, however, in 2009 (Fig. 3b), even as the number of new GWAS genes each year has grown (Fig. 3c). Thus, the proportion of hot genes that were recent GWAS hits has declined (Fig. 3d).

To further quantify the role of GWAS in creating hot genes, we used a logistic regression model (Table 2). Consistent with the overall probabilities (Fig. 3), this model showed that being a recent new GWAS hit was an important factor in determining whether a gene would be hot. The effect of being a GWAS hit, however, had a negative interaction with the year. In other words, the effect of GWAS on creating hot genes with exceptional publication records decreased with time.

**Table 2:** Logistic regression model for whether a gene exhibits a statistically significant excess in publications in a given year compared to the expectation of the Pfeiffer and Hoffmann model [38]

Predictor	Coefficient	Std. error	*p* value
log10(recent pubs)	3.881	0.068	<10^−32^
Year	−0.108	0.012	2 ×10^−18^
Recent GWAS	4.094	0.361	9×10^−30^
(Year × recent GWAS) interaction	−0.569	0.064	1×10^−18^

## Discussion

We analyzed the biomedical research publications to quantify the effect of genome-wide association studies on published scientific research. We found that even after the advent of GWAS, publications remain highly skewed toward Mendelian disease genes, with many complex disease genes receiving little attention (Fig. 1c). New complex disease genes identified by GWAS do receive additional study and subsequent publications (Fig. 2c), but that effect has declined (Fig. 2f, Table 1). Being newly associated with complex disease does increase a gene’s chance of becoming a “hot” gene, but this effect has also declined (Fig. 3d, Table 2). Together, our results suggest that GWAS have been successful in bringing research attention to novel genes involved in complex human disease, but this influence is waning.

Considering the overall distribution of biomedical publications, we found that GWAS have not reduced the inequality among human genes. The distribution of publications among human genes is characterized by a Gini coefficient of 0.73 in the post-GWAS era (Fig. 1a). By comparison, the Gini coefficient of money income among American households was 0.48 in 2016 [40] and among global households was 0.625 in 2013 [41]. The inequality of publications among genes is thus substantially greater than the inequality of income among households.

Focusing on individual genes, we found that association with complex disease via GWAS is correlated with an increase in subsequent publications (Fig. 2). Interestingly, the *p* value and estimated effect size of the association play a statistically insignificant role in determining the magnitude of that increase (Table 1 and Additional file 1: Table S1). We found a stronger effect on the subsequent publications for genes newly associated with metabolic disease (Additional file 1: Tables S3 and S4), perhaps reflecting its recent emphasis in public health [42]. We also found that association with complex disease via GWAS does raise the chances of a gene becoming an exceptionally studied “hot” gene (Fig. 3). But most dramatically, we found that the effects of new association via GWAS have declined over the past decade (Figs. 2f and 3d).

The direct results of a GWAS are associations of a disease with genetic variants, not with genes. For simplicity, we associated each variant with the closest gene, as long as that gene was within 500 kb. But many variants are regulatory, and gene regulation is complex, so some variants may actually most strongly affect other more distant genes [23]. Thus, some of the gene associations we study may be spurious. But this issue has existed since the advent of GWAS and has not changed markedly since. So, it cannot explain why the effect of GWAS on subsequent publications has declined over time. When studying the effects of genetic evidence on drug development, Nelson et al. [43] used a more complex approach for assigning variants to genes. They incorporated linkage disequilibrium and attempted to infer regulatory relationships using expression quantitative trait loci (eQTLs) and DNAse hypersensitivity sites. When we analyzed their collection of association data, we found similar results to our original analysis, although the effects were somewhat weaker (Additional file 1: Table S5 and Figure S7). In particular, we still found a negative relationship between the publication date of an association and its effect on the subsequent publications.

Our measures of scientific publications do not necessarily capture the full effects of GWAS on biomedical research. We considered studies of specific associated genes, but the broad insights GWAS has given into the genetic basis of human disease have substantially affected the biomedical research [10–12, 16]. Motivated by the example of complement factor H (Fig. 3a), we focused on the publications in a 3-year window following the GWAS. Some follow-up studies may take longer, but using a 5-year window does not change our qualitative conclusions (Additional file 1: Figure S8 and Tables S6 and S7). GWAS may also promote biomedical research in ways that do not involve new publications. For example, drugs with associated genetic evidence are more likely to progress along the development pipeline [43], suggesting that GWAS promote efficient drug development. More broadly, we focused on the associations with complex disease, the most common biomedical application of GWAS. But GWAS for drug response have already provided important guidance for personalized treatment [44]. Lastly, human GWAS have applications beyond health. For an evolutionary example, GWAS data have been used to detect adaptation in the human genome [45].

**Fig. 4:**
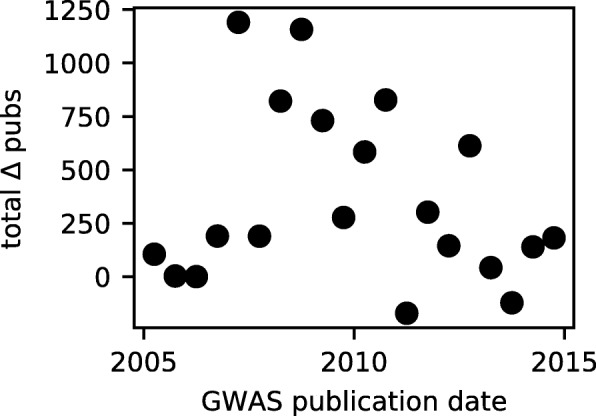
Total publication excess of new GWAS genes. For 6-month periods, plotted is the total publication excess (compared to control genes) of genes newly associated with complex disease via GWAS during each period

What explains the declining effect of GWAS on subsequent publications regarding newly associated genes? Perhaps early GWAS captured most genetic variants of large effect, so more recent studies find less compelling associations. But estimated effect size is not a strong predictor of subsequent publications (Table 1). Moreover, the typical estimated effect size of new associations has declined only modestly, and the absolute number of large-effect associations has grown (Additional file 1: Figure S9). Or perhaps journal publication criteria have changed over time, making GWAS less visible or follow-up studies more challenging to publish. The typical impact factor of journals GWAS are published in has declined slightly since the advent of GWAS (Additional file 1: Figure S10A). But the impact factor of the GWAS publication has only a weak effect on the publication excess of newly associated genes (Additional file 1: Figure S10B). When we included GWAS publication impact factor in our linear regression model, its effect was statistically significant but insufficient to explain the effect of publication date (Additional file 1: Table S8). Or perhaps researchers are spreading their effort among newly associated genes, so effects on individual genes have declined. But the summed publication excess over all genes newly associated with complex disease in a given time period has also declined over the past decade (Fig. 4). Or perhaps the availability of funding for follow-up studies has declined, as overall biomedical research funding has declined in both North America and Europe [46]. Or perhaps the capacity and interest to perform follow-up analyses has not kept pace with the “fire hose” of GWAS results [47]. Our data do not point toward a definitive explanation, and further investigation is needed to understand why recent GWAS promote less follow-up study on associated genes than early GWAS.

Over the past decade, GWAS have undeniably contributed greatly to biomedical knowledge [16]. The development of large-scale accessible databases of phenotypic and genotypic data, such as the UK Biobank [48], will fuel further contributions. But few GWAS results are directly medically actionable, so follow-up research is essential to translate novel associations into medical innovations. Our results suggest that the ability of GWAS to motivate published follow-up research on associated genes is declining. To maximize the positive impact of GWAS on human health, this trend must be understood and reversed.

## Materials and methods

### Publication data

We obtained Entrez GeneIDs for all 20,422 human protein-coding genes from NCBI Gene [36] on December 12, 2017. For all those genes, we collected PubMed identifiers of associated publications from NCBI Gene’s gene2pubmed file, downloaded December 12, 2017. This file contains both associations created manually during the curation of Gene References Into Function (GeneRIFs) and associations collected from organism-specific databases, Gene Ontology, and other curated data sources. We then obtained date information for each publication from PubMed, taking the earliest year between the reported year or EYear, using BioPython [49]. We followed a similar procedure for yeast genes. We obtained impact factor data from the 2016 InCites Journal Citation Reports [50].

### Disease data

To identify genes associated with Mendelian disease, we downloaded the Online Mendelian Inheritance in Man (OMIM) Gene Map of connections from genes to traits [37] on January 17, 2018. We filtered to keep only entries with a confidence code of “confirmed” and to ignore entries indicating a potentially spurious mapping or association with a non-disease trait. We further considered only entries with Entrez GeneIDs, to avoid ambiguity among gene names and aliases. This procedure yielded 1878 genes associated with disease traits. Of these, 1543 genes were associated with Mendelian but not complex multifactorial disease, 157 were associated with complex multifactorial but not Mendelian disease, and 178 were associated with both Mendelian and complex multifactorial disease.

To further identify genes associated with complex disease and to gather GWAS data, we used the January 1, 2017, release of NHGRI-EBI’s GWAS Catalog [5]. We filtered the catalog to remove non-disease traits, by keeping only entries that were children of the term “disease” (EFO0000408) in the Experimental Factor Ontology [51]. To connect associated variants with genes, we began with the Mapped Genes column in the catalog. We then connected each variant with its closest mapped gene, if that gene was within 500 kb. If a variant was within two overlapping genes, we connected with both genes. This procedure yielded 4069 genes associated with complex disease. To analyze the classes of disease, we used the children of the term “disease” in the Experimental Factor Ontology.

Our analysis of OMIM and the GWAS catalog yielded 5369 total disease-associated genes. Considering genes associated with only Mendelian disease in OMIM and not associated with disease through GWAS yielded 1126 Mendelian disease genes. Considering genes associated with only complex multifactorial disease in OMIM or associated with disease through GWAS yielded 3648 complex disease genes. The remaining 595 genes were associated with both Mendelian and complex disease.

Of the disease genes in the GWAS catalog, 2442 were first associated prior to 2015, so we could analyze three full years of publication data. For those genes, we identified odds ratios as reported effect sizes without units for variants that had a reported frequency of the risk allele. For our odds ratio analysis, we analyzed the 1327 genes for which an odds ratio was reported in the first year of GWAS association.

We also analyzed the association data of Nelson et al. [43]. They connected variants to genes using linkage disequilibrium, expression QTLs, and DNAse hypersensitivity. We filtered their Supplementary Data Set 1 to remove associations from OMIM, which may be Mendelian diseases. We also manually classified traits as disease or non-disease (Additional file 3), filtering out the non-disease traits.

### Control genes

For each of our 2442 GWAS genes, we identified its control gene as the non-GWAS gene with the closest number of total publications prior to the year the gene was first associated with complex disease. If multiple genes were tied for closest, we compared the previous year as well, continuing either until there was no ambiguity or until we reached 1950. For the 233 GWAS genes with ambiguous control genes, we compared subsequent publications between the GWAS gene and the average of the control genes.

### Publication rate model

We used the model of Pfeiffer and Hoffmann [38] to predict expected per-gene publication rates:

1 $$  \Delta P_{i,t+1} = \frac{k_{1} P^{*}_{t} + k_{2} P_{i,t}+k_{3}}{1+\left(P^{*}_{t}/P_{S}\right)^{\alpha}}.  $$

Here, *ΔP*_*i,t*+1_ is the predicted number of publications for gene *i* in year *t*+1, and *P*_*i,t*_ and $P^{*}_{t}$ are the cumulative number of publications in previous years for the gene and the average cumulative number of publications for all genes in the organism, respectively. The term in the denominator models saturation of publication rates. The three rate parameters, *k*_1_,*k*_2_, and *k*_3_, and the saturation parameters, *P*_*S*_ and *α*, were assumed to be identical for all genes. To fit the parameters to our data, we constructed a likelihood function by assuming that the number of publications each year for each gene was independently Poisson distributed with mean *ΔP*_*i,t*+1_ given by Eq. 1. We then maximized that likelihood with respect to the five model parameters, using publication data from 1950 to 2015 for all non-GWAS genes. The maximum-likelihood parameter values were *k*_1_=0.0214,*k*_2_=0.225,*k*_3_=0.00288,*P*_*S*_=24.1, and *α*=1.67. Five genes each had one publication prior to 1950 that was not included in the data fit.

To identify the years in which genes had significantly elevated publication rates, our null model was that publications were Poisson distributed with mean given by Eq. 1. Significant gene years were defined as those in which the probability of generating at least the observed number of publications was less than the Bonferroni-corrected significance cutoff 0.05/(*N*_*g*_*N*_*y*_). Here, *N*_*g*_=20,442 was the total number of genes considered, and *N*_*y*_=67 was the total number of years.

## Additional files

Additional file 1 Supplemental tables and figures. Supplemental **Tables S1–S8**, **Figure S1–S10**. (PDF 586 KB)

Additional file 2 Gene-years with exceptional publication activity. Gene-years with a statistically significant excess of publications relative to the prediction of the Pfeiffer and Hoffmann model. For GWAS disease genes, the date of the first GWAS to identify that gene is also recorded. (TSV 45 KB)

Additional file 3 Categorization of Nelson et al. traits. Traits from the association data of Nelson et al. [43], categorized as disease or non-disease. (TSV 16 KB)

## Abbreviations

GWAS: Genome-wide association study

## References

[1] Ozaki K, Ohnishi Y, Iida A, Sekine A, Yamada R, Tsunoda T (2002). Functional SNPs in the lymphotoxin- *α* gene that are associated with susceptibility to myocardial infarction. Nat Genet.

[2] Klein RJ, Zeiss C, Chew EY, Tsai JY, Sackler RS, Haynes C (2005). Complement factor H polymorphism in age-related macular degeneration. Science.

[3] DeWan A, Liu M, Hartman S, Zhang SSM, Liu DTL, Zhao C (2006). HTRA1 promoter polymorphism in wet age-related macular degeneration. Science.

[4] Burton PR, Clayton DG, Cardon LR, Craddock N, Deloukas P, Duncanson A (2007). Genome-wide association study of 14,000 cases of seven common diseases and 3000 shared controls. Nature.

[5] MacArthur J, Bowler E, Cerezo M, Gil L, Hall P, Hastings E (2016). The new NHGRI-EBI Catalog of published genome-wide association studies (GWAS Catalog). Nucleic Acids Res.

[6] Visscher PM, Brown MA, McCarthy MI, Yang J (2012). Five years of GWAS discovery. Am J Hum Genet.

[7] Manolio TA (2013). Bringing genome-wide association findings into clinical use. Nat Rev Genet.

[8] Balas EA, Boren SA, Bemmel J, McCray AT (2000). Managing clinical knowledge for health care improvement. Yearbook of Medical Informatics 2000: Patient-Centered Systems.

[9] Boyle EA, Li YI, Pritchard JK (2017). An expanded view of complex traits: from polygenic to omnigenic. Cell.

[10] Timpson NJ, Greenwood CMT, Soranzo N, Lawson DJ, Richards JB (2018). Genetic architecture: the shape of the genetic contribution to human traits and disease. Nat Rev Genet.

[11] Maurano MT, Humbert R, Rynes E, Thurman RE, Haugen E, Wang H (2012). Systematic localization of common disease-associated variation in regulatory DNA. Science.

[12] Sivakumaran S, Agakov F, Theodoratou E, Prendergast JG, Zgaga L, Manolio T (2011). Abundant pleiotropy in human complex diseases and traits. Am J Hum Genet.

[13] Pickrell JK, Berisa T, Liu JZ, Ségurel L, Tung JY, Hinds DA (2016). Detection and interpretation of shared genetic influences on 42 human traits. Nat Genet.

[14] Wray N, Goddard M, Visscher P (2007). Prediction of individual genetic risk to disease from genome-wide association studies. Genome Res.

[15] Chatterjee N, Shi J, García-Closas M (2016). Developing and evaluating polygenic risk prediction models for stratified disease prevention. Nat Rev Genet.

[16] Visscher PM, Wray NR, Zhang Q, Sklar P, McCarthy MI, Brown MA (2017). 10 years of GWAS discovery: biology, function, and translation. Am J Hum Genet.

[17] Dunham I, Kundaje A, Aldred SF, Collins PJ, Davis CA, Doyle F (2012). An integrated encyclopedia of DNA elements in the human genome. Nature.

[18] Kundaje A, Meuleman W, Ernst J, Bilenky M, Yen A, Roadmap Epigenomics Consortium (2015). Integrative analysis of 111 reference human epigenomes. Nature.

[19] Ardlie KG, DeLuca DS, Segrè AV, Sullivan TJ, Young TR, Gelfand ET (2015). The Genotype-Tissue Expression (GTEx) pilot analysis: multitissue gene regulation in humans. Science.

[20] Regev A, Teichmann SA, Lander ES, Amit I, Benoist C, Birney E (2017). The human cell atlas. Elife.

[21] Hirschhorn JN (2009). Genomewide association studies–illuminating biologic pathways. N Engl J Med.

[22] Ricigliano VAG, Umeton R, Germinario L, Alma E, Briani M, Di Segni N (2013). Contribution of genome-wide association studies to scientific research: a pragmatic approach to evaluate their impact. PLoS One.

[23] Edwards SL, Beesley J, French JD, Dunning M (2013). Beyond GWASs: Illuminating the dark road from association to function. Am J Hum Genet.

[24] Gallagher MD, Chen-Plotkin AS (2018). The post-GWAS Era: from association to function. Am J Hum Genet.

[25] Black JRM, Clark SJ (2016). Age-related macular degeneration: genome-wide association studies to translation. Genet Med.

[26] Duerr RH, Taylor KD, Brant SR, Rioux JD, Silverberg MS, Daly MJ (2006). A genome-wide association study identifies IL23R as an inflammatory bowel disease gene. Science.

[27] Cargill M, Schrodi SJ, Chang M, Garcia VE, Brandon R, Callis KP (2007). A large-scale genetic association study confirms IL12B and leads to the identification of IL23R as psoriasis-risk genes. Am J Hum Genet.

[28] Teng MWL, Bowman EP, McElwee JJ, Smyth MJ, Casanova JL, Cooper AM (2015). IL-12 and IL-23 cytokines: from discovery to targeted therapies for immune-mediated inflammatory diseases. Nat Med.

[29] Harold D, Abraham R, Hollingworth P, Sims R, Gerrish A, Hamshere ML (2009). Genome-wide association study identifies variants at CLU and PICALM associated with Alzheimer’s disease. Nat Genet.

[30] Mansiaux Y, Carrat F (2012). Contribution of genome-wide association studies to scientific research: a bibliometric survey of the citation impacts of GWAS and candidate gene studies published during the same period and in the same journals. PLoS ONE.

[31] Dolgin E (2017). The greatest hits of the human genome. Nature.

[32] Haynes WA, Tomczak A, Khatri P (2018). Gene annotation bias impedes biomedical research. Sci Rep.

[33] Isserlin R, Bader GD, Edwards A, Frye S, Willson T, Yu FH, Vol. 14. The human genome and drug discovery after a decade. Roads (still) not taken; 2011. http://arxiv.org/abs/1102.0448.

[34] Edwards AM, Isserlin R, Bader GD, Frye SV, Willson TM, Yu FH (2011). Too many roads not taken. Nature.

[35] Wilkening S, Chen B, Bermejo JL, Canzian F (2009). Is there still a need for candidate gene approaches in the era of genome-wide association studies?. Genomics.

[36] Brown GR, Hem V, Katz KS, Ovetsky M, Wallin C, Ermolaeva O (2015). Gene: a gene-centered information resource at NCBI. Nucleic Acids Res.

[37] Amberger JS, Bocchini CA, Schiettecatte F, Scott AF, Hamosh A (2015). OMIM.org: Online Mendelian Inheritance in Man (OMIM®;), an online catalog of human genes and genetic disorders. Nucleic Acids Res.

[38] Pfeiffer T, Hoffmann R (2007). Temporal patterns of genes in scientific publications. Proc Natl Acad Sci U S A.

[39] Fadista J, Manning AK, Florez JC, Groop L (2016). The (in)famous GWAS *P*-value threshold revisited and updated for low-frequency variants. Eur J Hum Genet.

[40] Semega JL, Fontenot KR, Kollar MA (2017). Income and poverty in the United States: 2016. U.S. Census Bureau, Current Population Reports, P60-259.

[41] World Bank (2016). Poverty and shared prosperity 2016: taking on inequality.

[42] Caballero B (2007). The global epidemic of obesity: an overview. Epidemiol Rev.

[43] Nelson MR, Tipney H, Painter JL, Shen J, Nicoletti P, Shen Y (2015). The support of human genetic evidence for approved drug indications. Nat Genet.

[44] Giacomini KM, Yee SW, Mushiroda T, Weinshilboum RM, Ratain MJ, Kubo M (2017). Genome-wide association studies of drug response and toxicity: an opportunity for genome medicine. Nat Rev Drug Discov.

[45] Berg JJ, Coop G (2014). A population genetic signal of polygenic adaptation. PLoS Genet.

[46] Chakma J, Sun GH, Steinberg JD, Sammut SM, Jagsi R (2014). Asia’s ascent: global trends in biomedical R&D expenditures. N Engl J Med.

[47] Hunter DJ, Kraft P (2007). Drinking from the fire hose-statistical issues in genomewide association studies. N Engl J Med.

[48] Sudlow C, Gallacher J, Allen N, Beral V, Burton P, Danesh J (2015). UK Biobank: an open access resource for identifying the causes of a wide range of complex diseases of middle and old age. PLoS Med.

[49] Cock PJA, Antao T, Chang JT, Chapman Ba, Cox CJ, Dalke A (2009). Biopython: freely available Python tools for computational molecular biology and bioinformatics. Bioinformatics.

[50] Clarivate Analytics. 2016 Journal Citation Reports *Ⓡ*; 2017. http://ipscience-help.thomsonreuters.com/incitesLiveJCR/JCRGroup/howtoCiteJCR/version/10.

[51] Malone J, Holloway E, Adamusiak T, Kapushesky M, Zheng J, Kolesnikov N (2010). Modeling sample variables with an Experimental Factor Ontology. Bioinformatics.

