# Supplementary material for: The impact of genome-wide association studies on biomedical research publications
Source: Hum Genomics. 2018 Aug 13;12:38. doi: 10.1186/s40246-018-0172-4 (PMC6090631; doi:10.1186/s40246-018-0172-4)
Supplement: Supplementary file 1 — Supplemental tables and figures. Supplemental Tables S1–S8, Figure S1–S10. (PDF 586 KB) [file 40246_2018_172_MOESM1_ESM.pdf]

predictor	coefficient	std. error	p-value
$\log_{10}$(recent pubs)	-1.547	0.548	0.005
-$\log_{10}$(p-value)	-0.011	0.029	0.690
estimated odds ratio	0.136	0.187	0.468
GWAS pub. date	-1.233	0.163	$< 10^{-12}$

Table S1: As in Table 1, linear regression model for the normalized publication excess of new GWAS genes ($N = 517$), but restricted to genes for which the first reported association had $p < 10^{-8}$.

predictor	coefficient	std. error	p-value
$\log_{10}$(recent pubs)	-1.985	0.897	0.028
-$\log_{10}$(p-value)	-0.007	0.040	0.858
estimated odds ratio	0.141	0.238	0.553
GWAS pub. date	-1.446	0.274	$< 10^{-6}$

Table S2: As in Table S1, but further restricting the analysis to cases in which the lower bound of the 95% confidence interval on the odds ratio was greater than 1.1 ($N = 296$).

predictor	# of genes	coefficient	std. error	p-value
$\log_{10}$(recent pubs)		-0.740	0.284	0.009
-$\log_{10}$(p-value)		0.038	0.019	0.044
estimated odds ratio		0.031	0.064	0.629
GWAS pub. date		-0.756	0.083	10^{-19}
brain aneurysm	4	-1.816	2.844	0.523
cardiovascular disease	95	-0.578	0.793	0.466
chemotherapy-induced alopecia	8	1.498	2.280	0.511
digestive system disease	140	0.439	0.680	0.518
endocrine system disease	10	3.004	1.941	0.122
eye disease	7	1.113	2.192	0.612
genetic disorder	40	-0.542	1.071	0.613
head and neck disorder	24	0.263	1.229	0.830
immune system disease	324	-0.760	0.515	0.141
infectious disease	35	0.333	1.103	0.763
kidney disease	29	-1.265	1.189	0.287
liver disease	16	-0.394	1.504	0.793
mental or behavioural disorder	139	0.180	0.693	0.795
metabolic disease	138	1.783	0.690	**0.010**
neoplasm	263	0.025	0.624	0.968
nervous system disease	231	0.579	0.599	0.333
reproductive system disease	27	1.119	1.201	0.352
respiratory system disease	43	-0.136	0.982	0.89
skeletal system disease	74	1.133	0.826	0.171
skin disease	37	-0.203	1.056	0.848

Table S3: Effects of disease class on publication excess. We added parameters to the linear model designating whether or not a gene was first associated with a particular disease class. Only for metabolic disease do we see a statistically significant effect on publication excess. The second column shows the number of genes first associated with each disease class.

predictor	# of genes	coefficient	std. error	p-value
type I diabetes mellitus	32	-0.378	1.154	0.743
type II diabetes mellitus	62	6.024	0.887	$\mathbf{10^{-11}}$
obesity	33	5.690	1.106	$\mathbf{10^{-7}}$

Table S4: Effects of different metabolic diseases on publication excess. We modified our linear model to remove the general metabolic disease parameter and add the three specific diseases with a large number of genes. Remaining model parameters (not shown) are similar to the regression in Table S3.

predictor	coefficient	std. error	p-value
$\log_{10}$(recent pubs)	-0.119	0.235	0.614
-$\log_{10}$(p-value)	-0.007	0.008	0.393
GWAS pub. date	-0.448	0.073	10^{-9}

Table S5: As in Table 1, linear regression model for the normalized publication excess of newly associated genes ($N = 1,861$), but using the data of Nelson et al. (2015). Note that they did not collect effect size information. The negative effect of publication date remains, although it is somewhat weaker.

predictor	coefficient	std. error	p-value
$\log_{10}$(recent pubs)	-0.558	0.524	0.287
-$\log_{10}$(p-value)	0.053	0.036	0.143
estimated odds ratio	-0.008	0.108	0.944
GWAS pub. date	-1.554	0.191	10^{-15}

Table S6: As in Table 1, linear regression model for the normalized publication excess of new GWAS genes ($N = 873$), but with a five-year range for calculating Δ pubs and recent pubs.

predictor	coefficient	std. error	p-value
$\log_{10}$(recent pubs)	4.081	0.070	$< 10^{-32}$
year	-0.100	0.013	10^{-15}
recent GWAS	4.124	0.372	10^{-28}
(year $\times$ recent GWAS) interaction	-0.575	0.066	10^{-17}

Table S7: As in Table 2, logistic regression model for whether a gene exhibits a statistically significant excess in publications in a given year, compared to the expectation of the Pfeiffer and Hoffmann model, but with a 5-year range for defining recent pubs and GWAS.

predictor	coefficient	std. error	p-value
$\log_{10}$(recent pubs)	-0.807	0.291	0.006
-$\log_{10}$(p-value)	0.026	0.022	0.235
estimated odds ratio	0.050	0.062	0.420
-$\log_{10}$(impact factor)	0.422	0.192	0.028
GWAS pub. date	-0.706	0.081	10^{-17}

Table S8: As in Table 1, linear regression model for the normalized publication excess of newly associated genes ($N = 1,191$), but including the effect of impact factor for the GWAS publication.

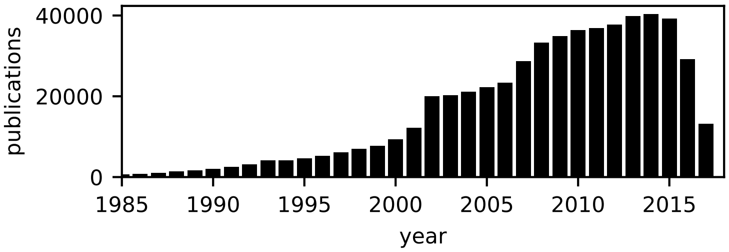

Figure S1: Distribution of analyzed publications by year published.

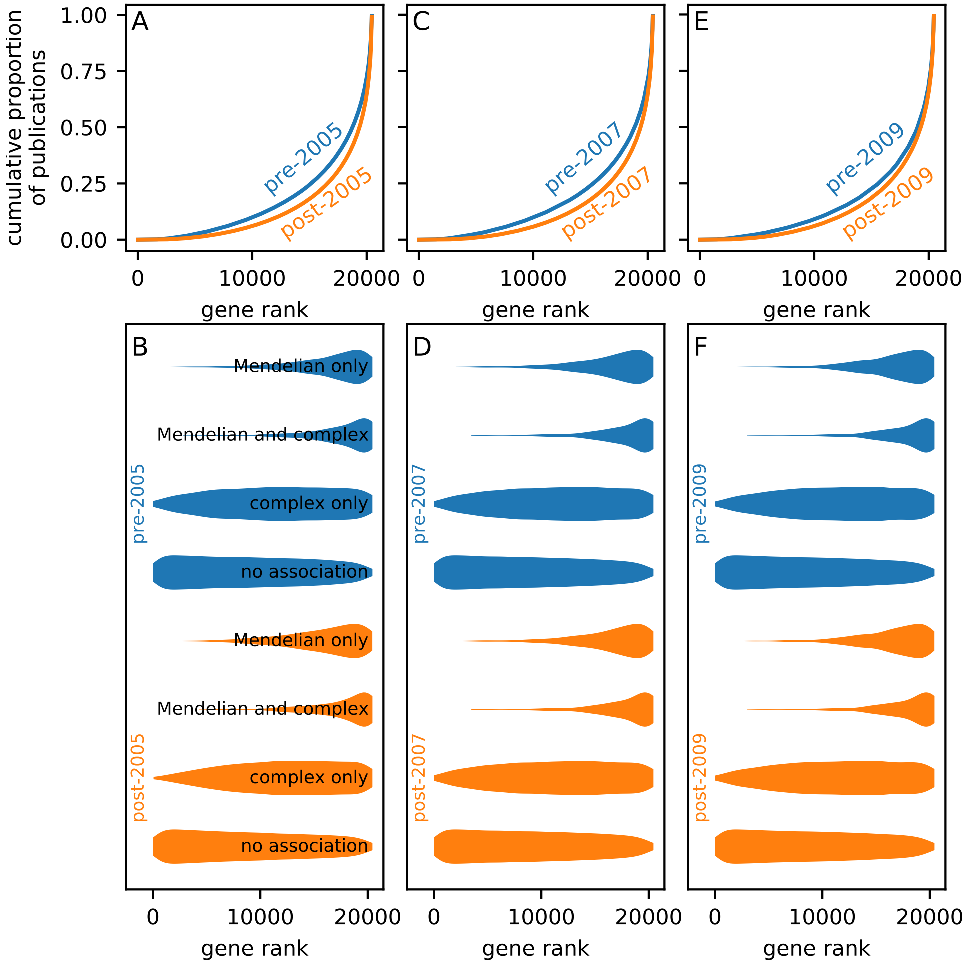

Figure S2: A&B: Reproduction of Fig. 1C&D. C-F: As in A&B, but using 2007 or 2009 as the cutoff year between pre- and post-GWAS eras.

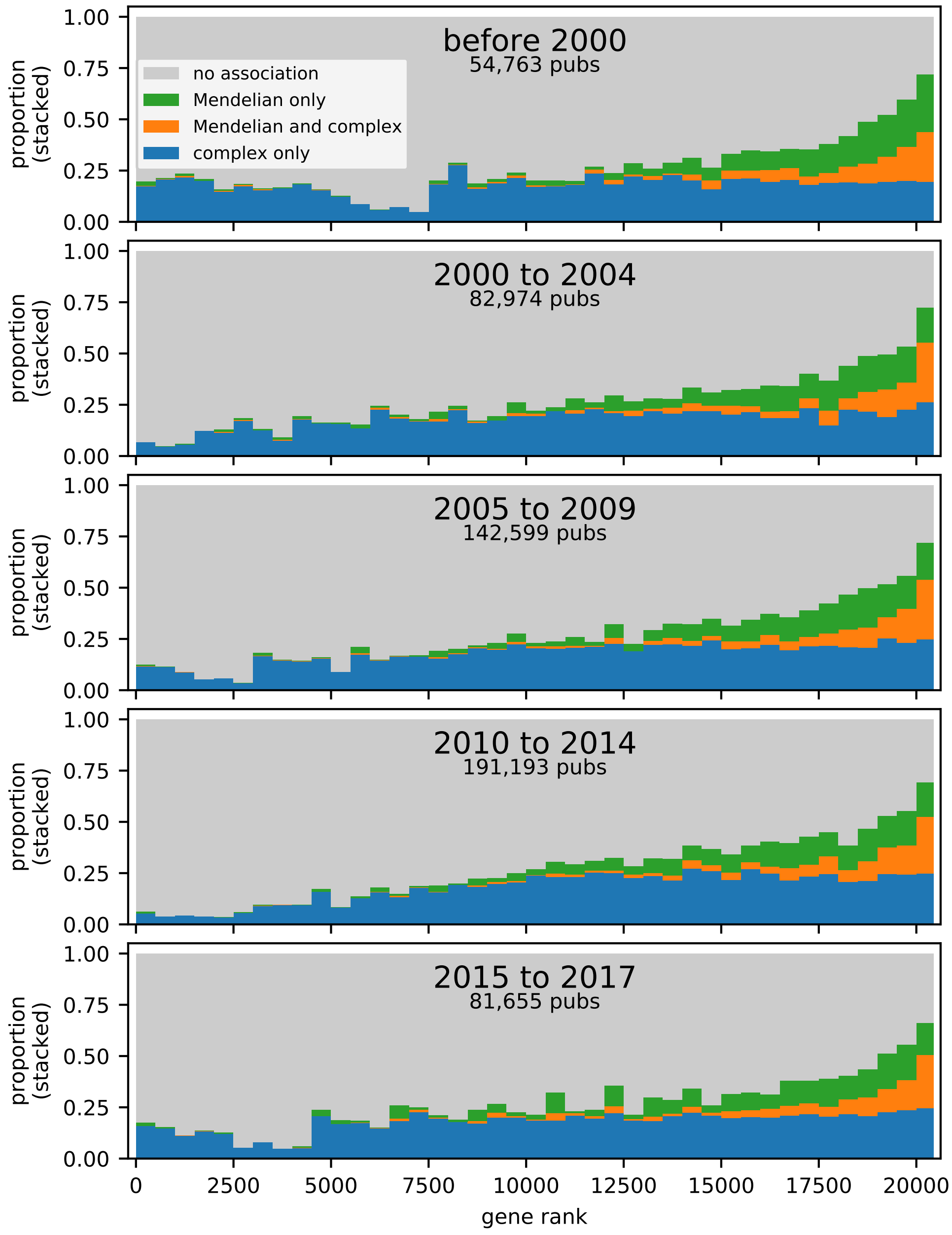

Figure S3: Distributions of publication gene ranks for genes of different disease classes for various time intervals. Each panel is a stacked bar plot that shows the proportion of genes that are in a given disease class within each bin of gene ranks. Genes are ranked separately for each panel, considering only publications from the years indicated.

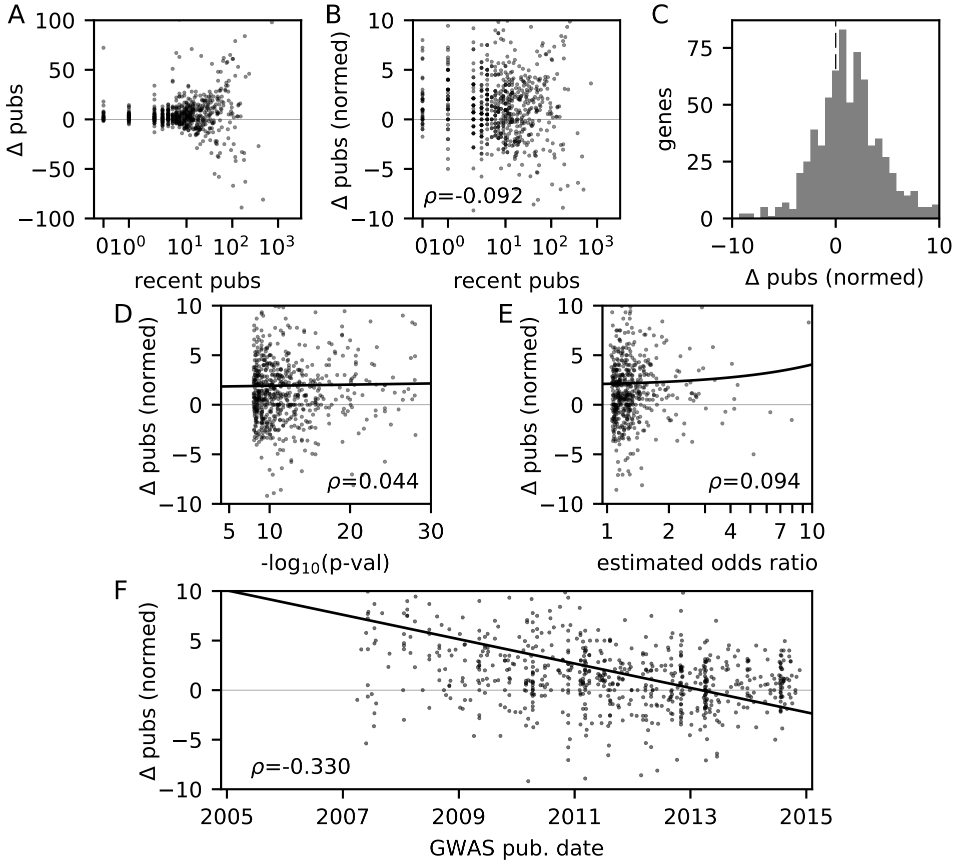

Figure S4: As in Fig. 2, but restricting the analysis to genes for which the first reported association had $p < 10^{-8}$.

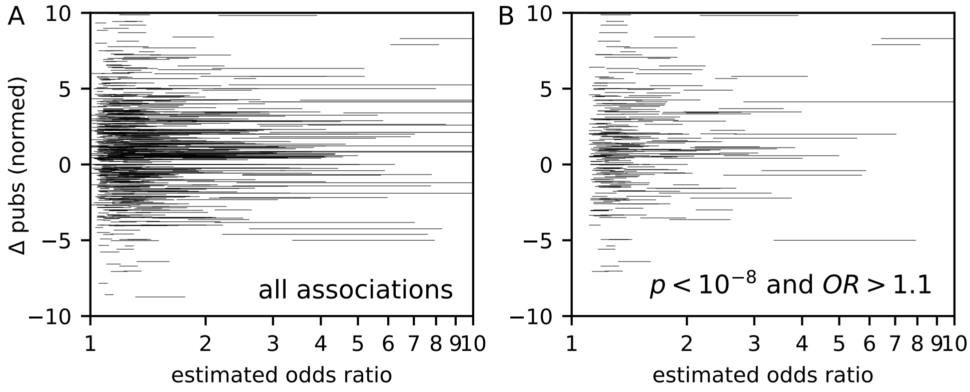

Figure S5: Publication excess versus reported odds ratio, with uncertainties. A: As in Fig. 2E, but now showing 95% confidence intervals on the odds ratio, for the 1,094 associations with intervals reported in the GWAS catalog. B: As in panel A, but restricted to associations for which $p < 10^{-8}$ and the lower bound of the 95% confidence interval on the odds ratio was > 1.1.

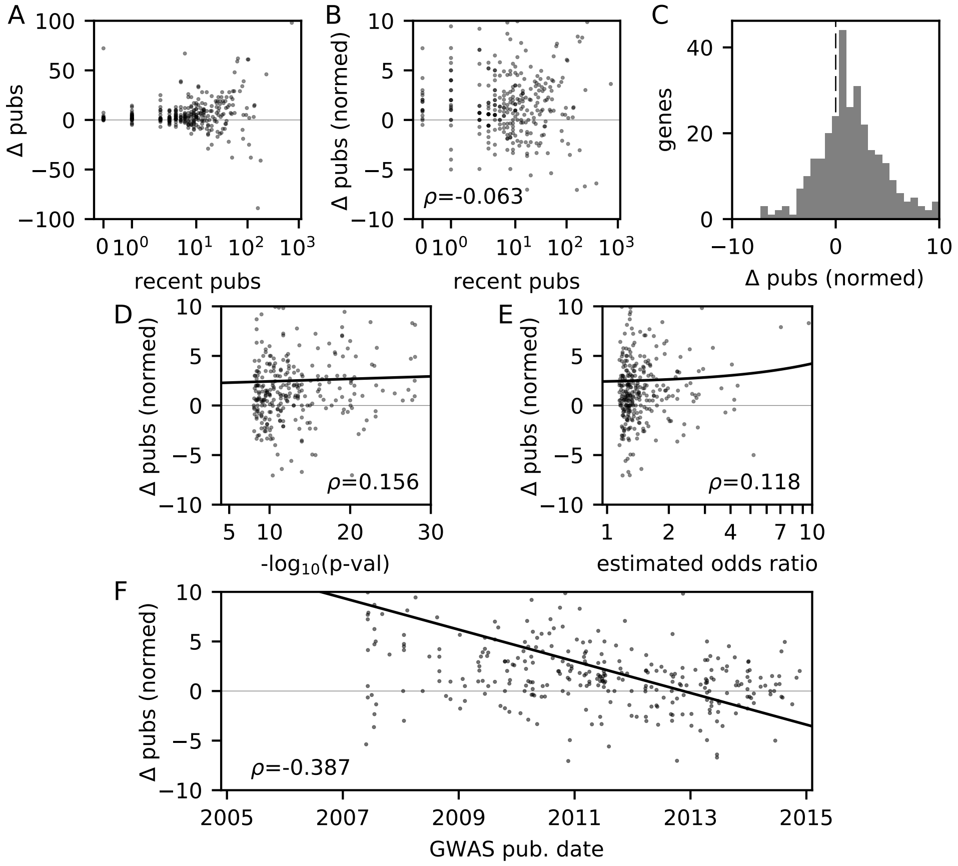

Figure S6: As in Fig. 2, but restricting the analysis to genes for which the first reported association had $p < 10^{-8}$ and the lower bound on the 95% confidence interval for the estimated effect size was > 1.1.

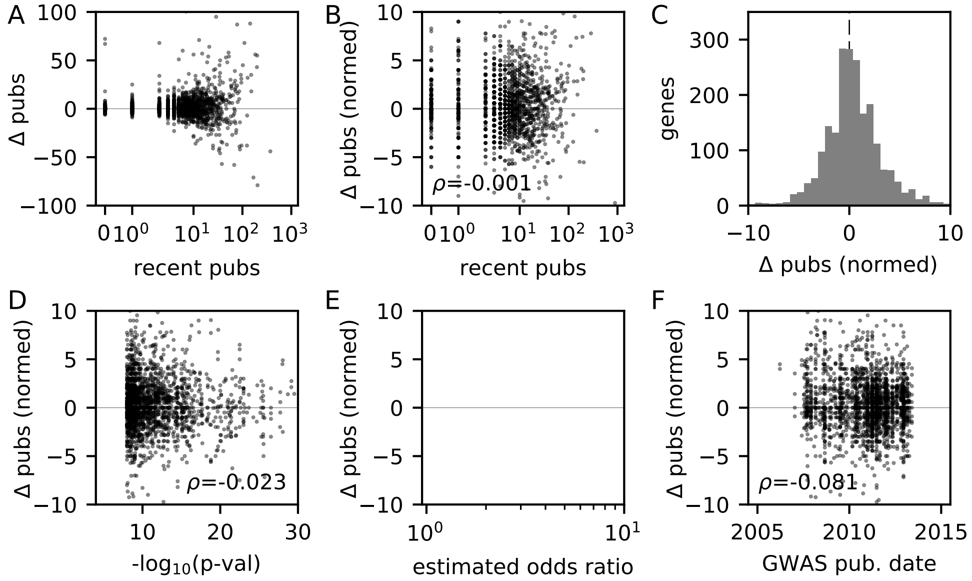

Figure S7: Results using association data from Nelson et al. (2015). Panels as in Fig. 2, but panel E is empty because Nelson et al. did not collect odds ratios. Broadly, the effects are similar to those in our collection of data, but weaker. For the Nelson et al. data, the mean normalized publication excess is 0.71, compared to 1.24 in our collection of data.

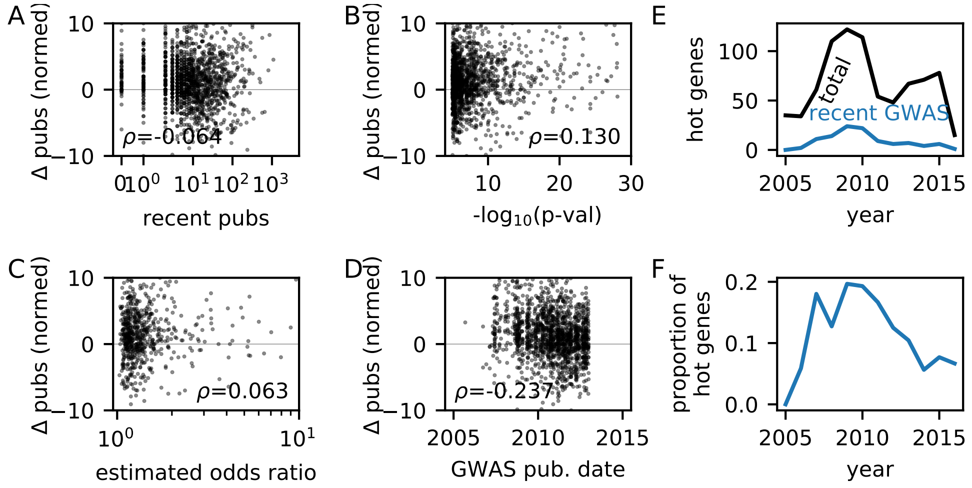

Figure S8: Results using a 5 year window for counting publications and defining recent GWAS. A-D: As in Fig. 2. E&F: As in Fig. 3.

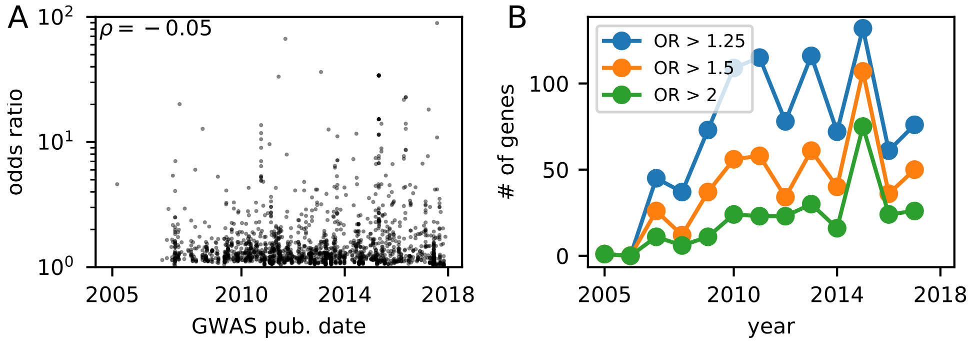

Figure S9: Trend in estimated effect size of new GWAS genes. A: For genes newly associated with complex disease, the typical estimated odds ratio has slightly declined over time (Spearman rank correlation $\rho = -0.05$, $p \sim 0.033$, $N = 1,812$). B: The number of new GWAS genes with large estimated odds ratios has not, however, declined with time.

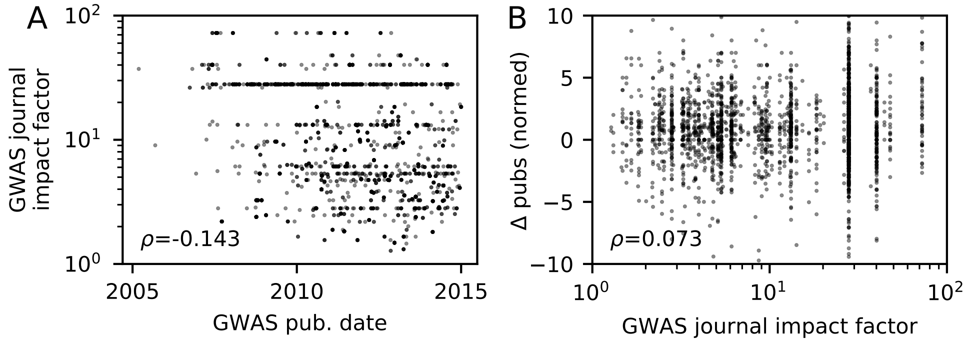

Figure S10: Effect of journal impact factor. A: The typical impact factor of GWAS publications has declined slightly since the advent of GWAS. B: The journal impact factor of the GWAS publication is weakly correlated with publication excess of the reported associated genes.
